# Virus in infectious uveitis: bibliometric analysis and a clinical study

**DOI:** 10.3389/fmicb.2025.1588195

**Published:** 2025-08-07

**Authors:** Junhui Shen, Jinfeng Kong, Yufeng Xu, Yanyan Hu, Lei Feng

**Affiliations:** ^1^Eye Center, Second Affiliated Hospital, School of Medicine, Zhejiang University, Hangzhou, China; ^2^Key Laboratory of Ophthalmology of Zhejiang Province, Hangzhou, China; ^3^Department of Clinical Laboratory, Second Affiliated Hospital, School of Medicine, Zhejiang University, Hangzhou, China

**Keywords:** infectious uveitis, bibliometric analysis, metagenomic next-generation sequencing, virus, intraocular fluid

## Abstract

**Introduction:**

In recent years, advancements in detection technology have led to increased research interest in viral uveitis.

**Methods:**

This study conducted a comprehensive analysis, comprising a bibliometric examination of literature on virus and infectious uveitis and a retrospective study focusing on infectious uveitis. The bibliometric analysis aimed to elucidate past and emerging trends in this field over several decades. In the retrospective study, intraocular fluid samples were collected from 73 patients suspected of having infectious uveitis for metagenomic next-generation sequencing (mNGS), with 29 samples also subjected to microbiological culture.

**Results:**

Analysis of the literature revealed a steady rise in annual publications on virus and infectious uveitis from 1990 to 2021, reaching a peak in 2021. The United States emerged as the most prolific contributor, with significant collaborative relationships with other nations. Keywords were clustered into five categories, covering diagnostic criteria, diagnostic tools, clinical manifestations, epidemiology, and etiology of viral uveitis. Interestingly, research focus shifted from predominant viral types and serodiagnosis towards intraocular fluid testing. mNGS demonstrated a notably higher positivity rate (73.97%) compared to culture (3.45%), identifying various pathogens including viruses, bacteria, fungi, *Toxoplasma gondii*, and *Rickettsia felis*. Varicella-Zoster Virus, Epstein–Barr Virus, *Klebsiella pneumoniae*, and Torque Teno Virus were among the most common pathogens detected. Additionally, coexisting microorganisms such as Torque Teno Virus and Epstein–Barr Virus were identified.

**Conclusion:**

Viral uveitis has consistently garnered research attention, with future directions likely focusing on virus types and diagnostic tools. Viruses are the main causative microorganisms of infectious uveitis. The high efficacy of mNGS in identifying diverse pathogens from minute volumes of intraocular fluid samples highlights its pivotal role in diagnosing infectious uveitis.

## Background

Uveitis represents a significant contributor to ocular morbidity, accounting for 5–10% of visual impairment cases globally (Tsirouki et al., 2018). Infectious uveitis is a potentially sight-threatening condition characterized by intraocular inflammation resulting from various infectious agents, such as viruses, bacteria, fungi, or parasites (Shen et al., 2022). Early diagnosis and rapid treatment are crucial for controlling intraocular inflammation.

With the improvement of detection technology in recent years, research on viral uveitis is increasing. There have been a few reports of Epstein–Barr Virus (EBV) infection associated with panuveitis (Silpa-Archa et al., 2022). Varicella Zoster Virus (VZV) is the most common virus found in atypical necrotizing retinitis (Lee et al., 2017). Cytomegalovirus retinitis often occurs in individuals with acquired immunodeficiency syndrome (AIDS) or immune suppression (Xu et al., 2024). However, there is a lack of systematic quantitative research analysis to date. Bibliometrics, as a multi-system discipline that integrates mathematics, statistics, philology, and other disciplines, mainly utilizes mathematical and statistical methods to quantitatively analyze papers, making the characteristics and development trends intuitively visible by clustering literature and building knowledge graphs (Ying et al., 2023). At present, no bibliometric studies that have been performed to investigate the virus and infectious uveitis.

In recent years, pathogens have undergone several changes and trends, driven by various factors including environmental changes, human behavior, globalization, and advancements in science and technology. New pathogens have emerged or re-emerged, posing significant public health challenges. Examples include the Ebola virus, Zika virus, Middle East Respiratory Syndrome Coronavirus (MERS-CoV), and Severe Acute Respiratory Syndrome Coronavirus 2 (SARS-CoV-2) (Deng et al., 2020).

Metagenomic next-generation sequencing (mNGS) has revolutionized the field of infectious disease diagnostics, including its application in understanding and managing infectious uveitis. mNGS can simultaneously detect multiple pathogens in a single sample, making it a valuable tool for identifying the specific microorganism in a short time. mNGS can help identify these uncommon infectious agents by analyzing the genomic characterization of the pathogens (Valdes et al., 2020). For example, Lee and collaborators reported the detection of the torque teno virus genome in samples of culture-negative endophthalmitis using mNGS (Lee et al., 2015). Polymerase chain reaction (PCR) can be used to detect viral infections, however, compared with mNGS, PCR may be less adept at identifying emerging or less prevalent pathogens (Dutta Majumder et al., 2023).

Therefore, in this article, we aim to examine the publication trend and relevance of scientific production in uveitis and viruses, providing a bibliographic profile of the publications in the relevant databases to examine the trends over times. Furthermore, we will investigate the utilization of mNGS in suspected cases of infectious uveitis in Chinese patients, and analyze the distribution of infectious uveitis pathogens in recent years.

## Methods

### Search strategy and data retrieval

A systematic search on Web of Science Core Collection (WoSCC) database was conducted on Dec 9, 2023. The search formula was as followed: [TS = (infectious uveitis) AND TS = (virus)], the type of documents was set to “articles,” the type of language was set to “English.” The time span was set from the beginning of the database to Dec 9, 2023.

### Analytic methods and software used

We used three bibliometric analysis software to extract bibliographic information from downloaded files to construct visualized network maps. VOSviewer (version 1.6.17) is a bibliometric analysis software that can extract the key information from plentiful publications (van Eck and Waltman, 2010), which is used to build collaboration, co-citation and co-occurrence networks. In our study, we use the software mainly to complete the following analysis: country and institution analysis and keyword co-occurrence analysis. CiteSpace (version 5.7.2. R1) is a widely used tool for visual exploration of scientific literature developed by Professors Chen C (Synnestvedt et al., 2005). In our study, CiteSpace was applied to analyze keywords with Citation Bursts (Chen, 2004). The R package “bibliometrix” (version 3.2.1) was applied for a thematic evolution analysis and to construct a global distribution network of publications (Aria et al., 2017). Additionally, Microsoft Office Excel 2019 was used to conduct quantitative analysis of publication. Continuous data are shown as the mean ± standard deviation (SD).

### Subjects

Patients diagnosed with suspected infectious uveitis between July 2021 and July 2023 in Second Affiliated Hospital of Zhejiang University were enrolled. This retrospective, cross-sectional study was approved by the Institutional Review Board of the Second Affiliated Hospital of Zhejiang University School of Medicine.

### Inclusion and exclusion criteria

Suspected infectious uveitis cases were included. We excluded patients with autoimmune uveitis and masquerading syndrome. Patients should not respond to conventional steroid therapy. The consent procedure and study protocol followed the tenets of the Declaration of Helsinki. Written informed consent was obtained.

### Clinical information collection

Detailed clinical data, including patient age, gender, best corrected visual acuity (BCVA), intraocular pressure (IOP), characteristics of ocular disease, and treatment condition were collected. Clinical diagnosis of suspected infectious uveitis based on the history of risk factors, vision decrease, anterior chamber, and vitreous hypopyon. Other testing methods were also conducted to identify the etiology of disease, including the T-SPOT test for suspected tuberculosis infection, smear and culture of suspected bacteria or fungi. The final diagnosis was confirmed based on the clinical signs, the laboratory examinations, and the response to the treatment. Intraocular fluid is obtained either through anterior chamber puncture or through vitrectomy. If both eyes are affected, we will choose the more serious eye for testing.

### Culture of suspected bacteria or fungi

Inoculate the sterile specimens collected above in a sterile environment onto a suitable culture medium, and then place them in a carbon dioxide incubator with set temperature and carbon dioxide concentration according to the culture conditions. Regularly observe the growth of microorganisms, isolate and purify them, and confirm them through morphological observation, physiological and biochemical tests.

### Metagenomic next-generation sequencing and analysis

Undiluted vitreous or aqueous humor (0.3 mL) was collected from patients during operation. DNA was extracted using TIANamp Micro DNA Kit (DP316, TIANGEN BIOTECH, Beijing, China) following the manufacturer’s operational manual. The extracted DNA was amplified for the construction of DNA libraries. DNA libraries were created through processes including DNA fragmentation, end repair, adapter ligation, and PCR amplification. The quality of these DNA libraries was assessed using the Agilent 2,100 system. Libraries that met quality standards were combined and subjected to DNA Nanoball (DNB) formation, followed by sequencing on the MGISEQ-2000 platform. Low-quality reads were filtered out, and human host sequences were computationally subtracted by aligning them to the human reference genome (hg19) using the Burrows-Wheeler Alignment tool. The remaining data, after removal of low-complexity reads, were classified by aligning them simultaneously to the Pathogens Metagenomics Database (PMDB), which includes bacteria, fungi, viruses, and parasites. The reference databases for classification were obtained from NCBI.^1^ For mNGS detection, negative and positive control were used, and analyzed with the same procedure as clinical samples.

## Results

### Global annual publications

**Figure 1:**
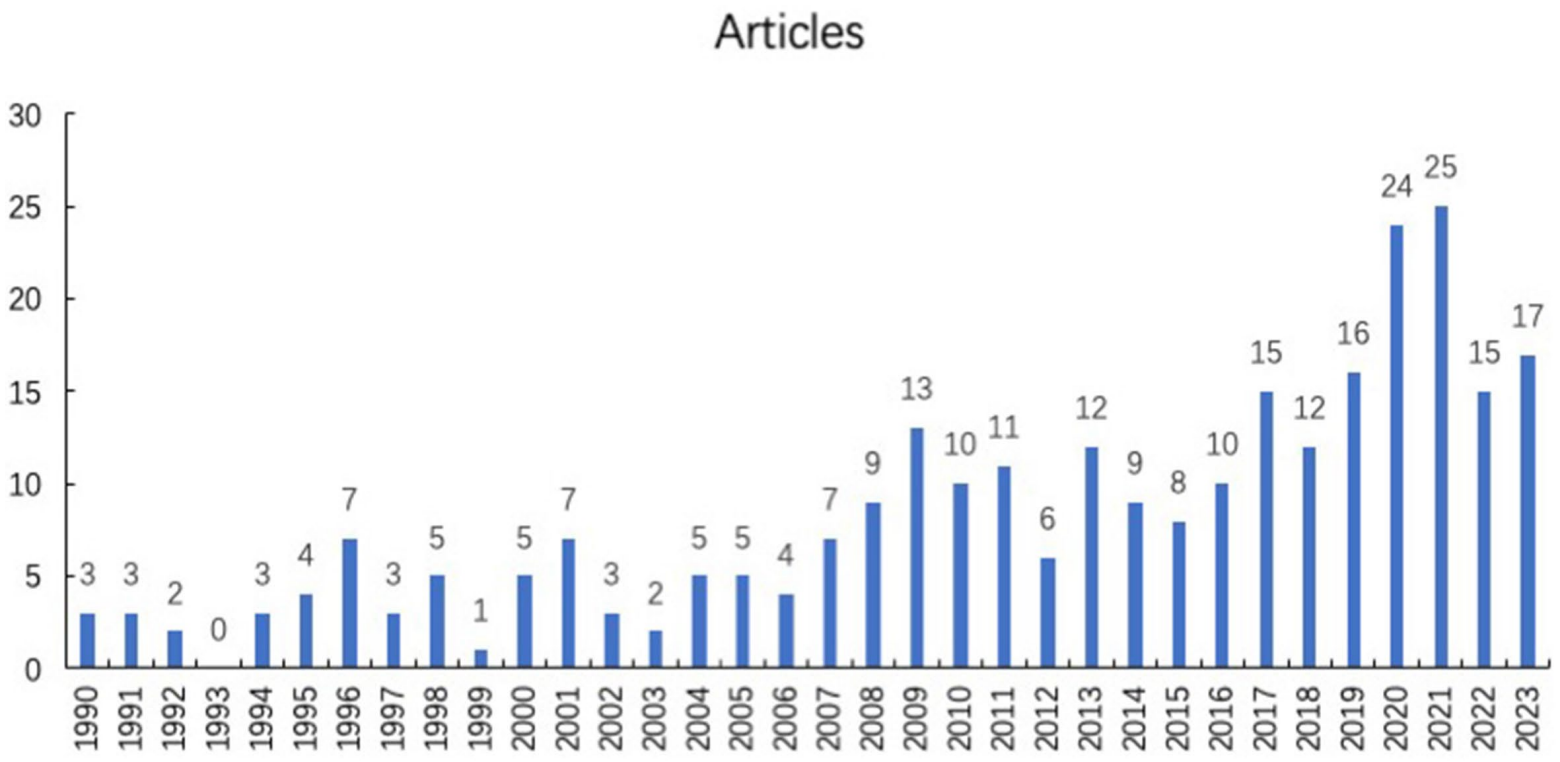
Annual scientific publication of virus and infection uveitis articles.

A total of 281 publications were acquired from the database by using our search strategy. As shown in Figure 1, from 1990 to 2021, the annual publication number was risen steadily. There were a certain number of articles about virus and infectious uveitis, which means that this topic was concerned constantly. Since 2016, more than 10 research papers on this topic have been published every year. Among them, 24 and 25 papers were published in 2020 and 2021, respectively. In the past 3 years, the number of publications of infectious uveitis has increased significantly and promptly. With the COVID-19 epidemic hitting all over the world, the role of virus and the pathogenesis of infectious uveitis has been paid more and more attention.

### Top countries/regions contributing to global publications

**Figure 2:**
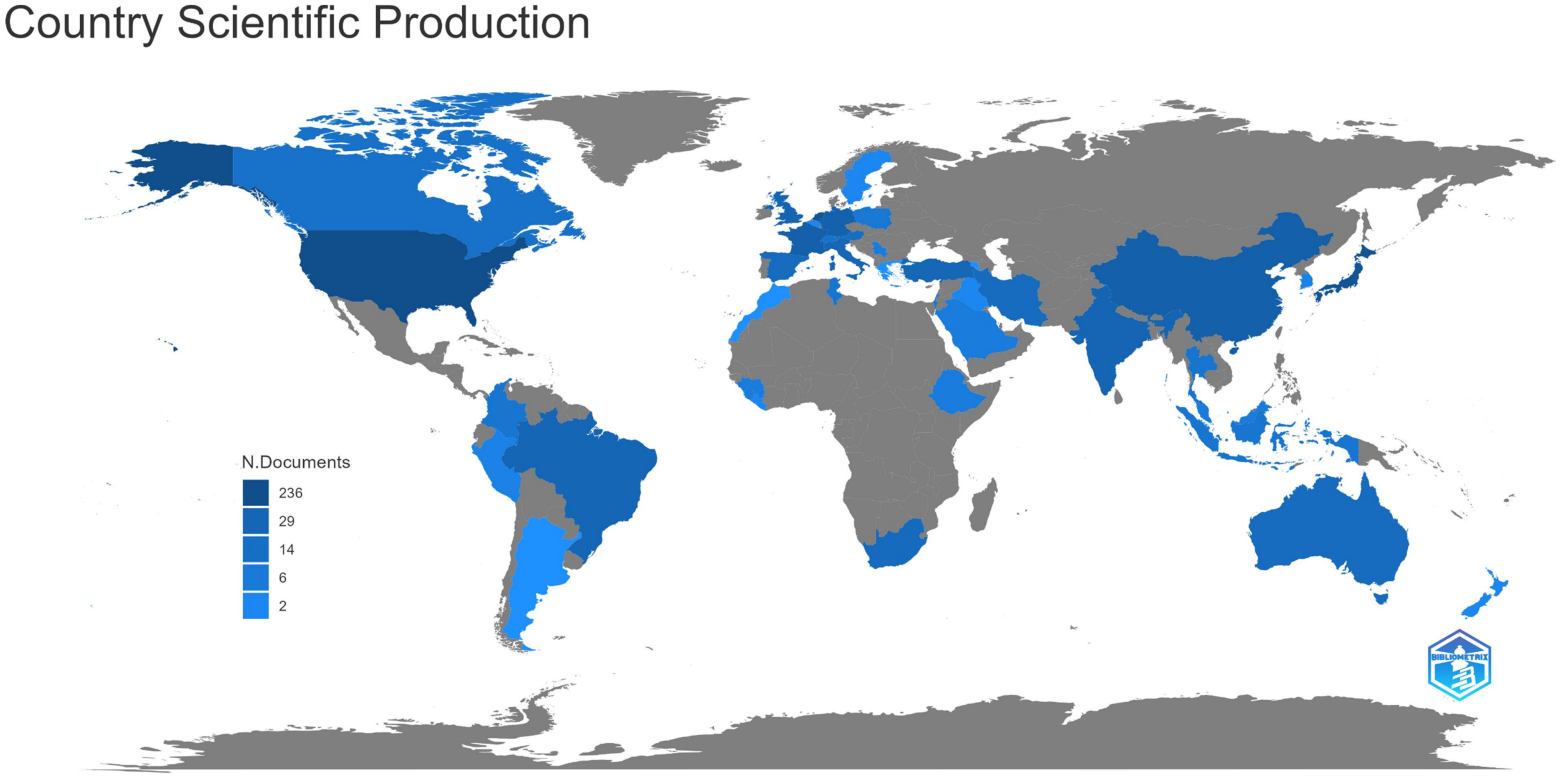
The distribution of countries/regions with published literature of virus and infectious uveitis.

The distribution of countries/regions with published literature is shown in Figure 2, and the countries/regions marked in blue are those with published relevant literature. Among them, the United States published the most articles (64, 22.8%), followed by Japan (30, 10.7%), France (25, 8.9%), the Netherlands (22, 7.8%), Germany (17, 6.0%), China (14, 5.0%), India (9, 3.2%), Brazil (8, 2.8%), and the United Kingdom (8, 2.8%). It can be seen that countries with a large number of publications are mostly developed countries or densely populated countries.

### Cooperation network analyses of country/region

**Figure 3:**
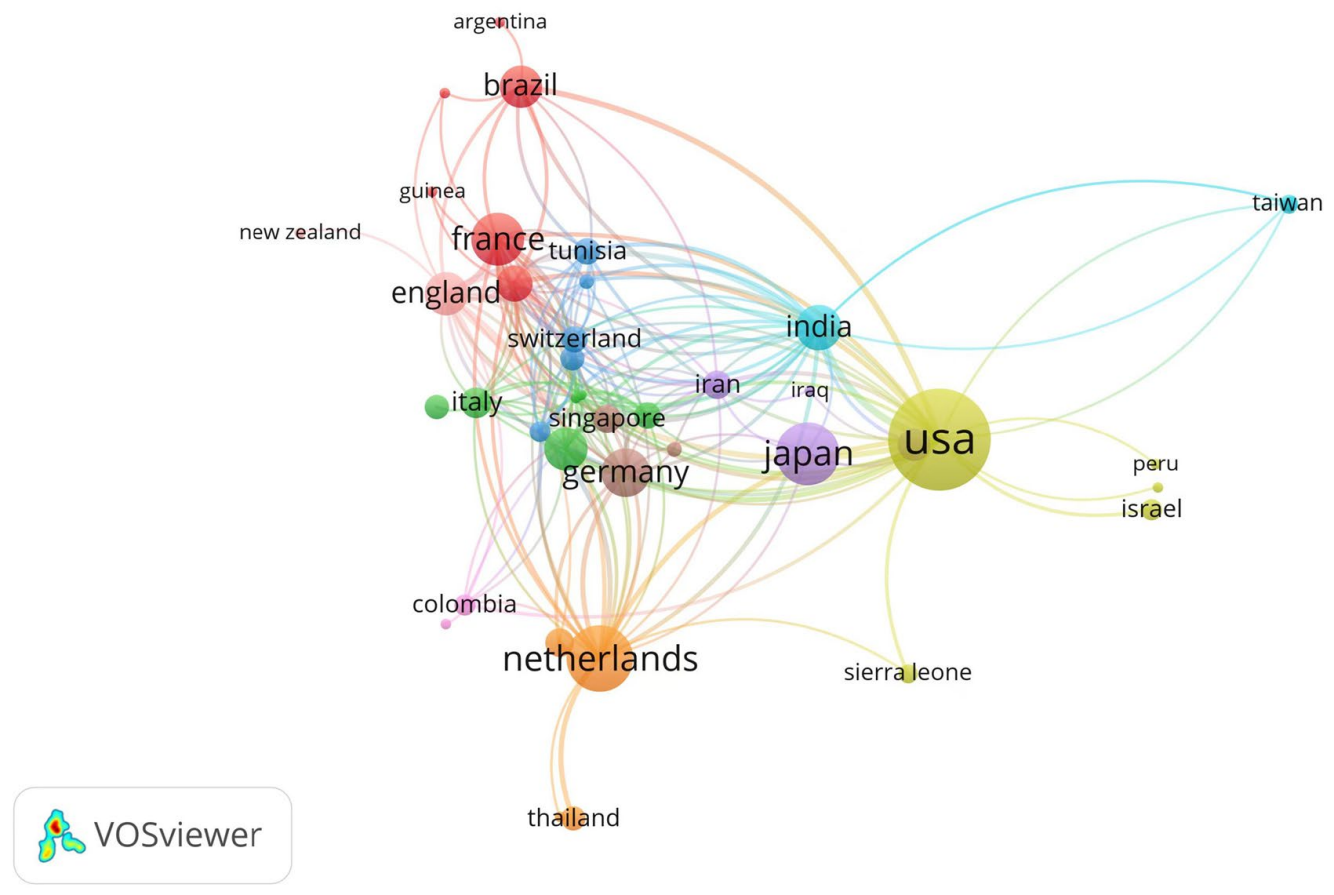
A representative figure demonstrating the publishing collaborations between various countries.

As shown in Figure 3, the size of each country’s name corresponds to the number of articles published by that country, while the thickness of the connecting lines indicates the level of collaboration between each pair of countries. It’s evident that the United States, with a substantial volume of publications, engages in more frequent collaboration with other nations.

### The co-occurrence analysis of the top keywords

**Figure 4:**
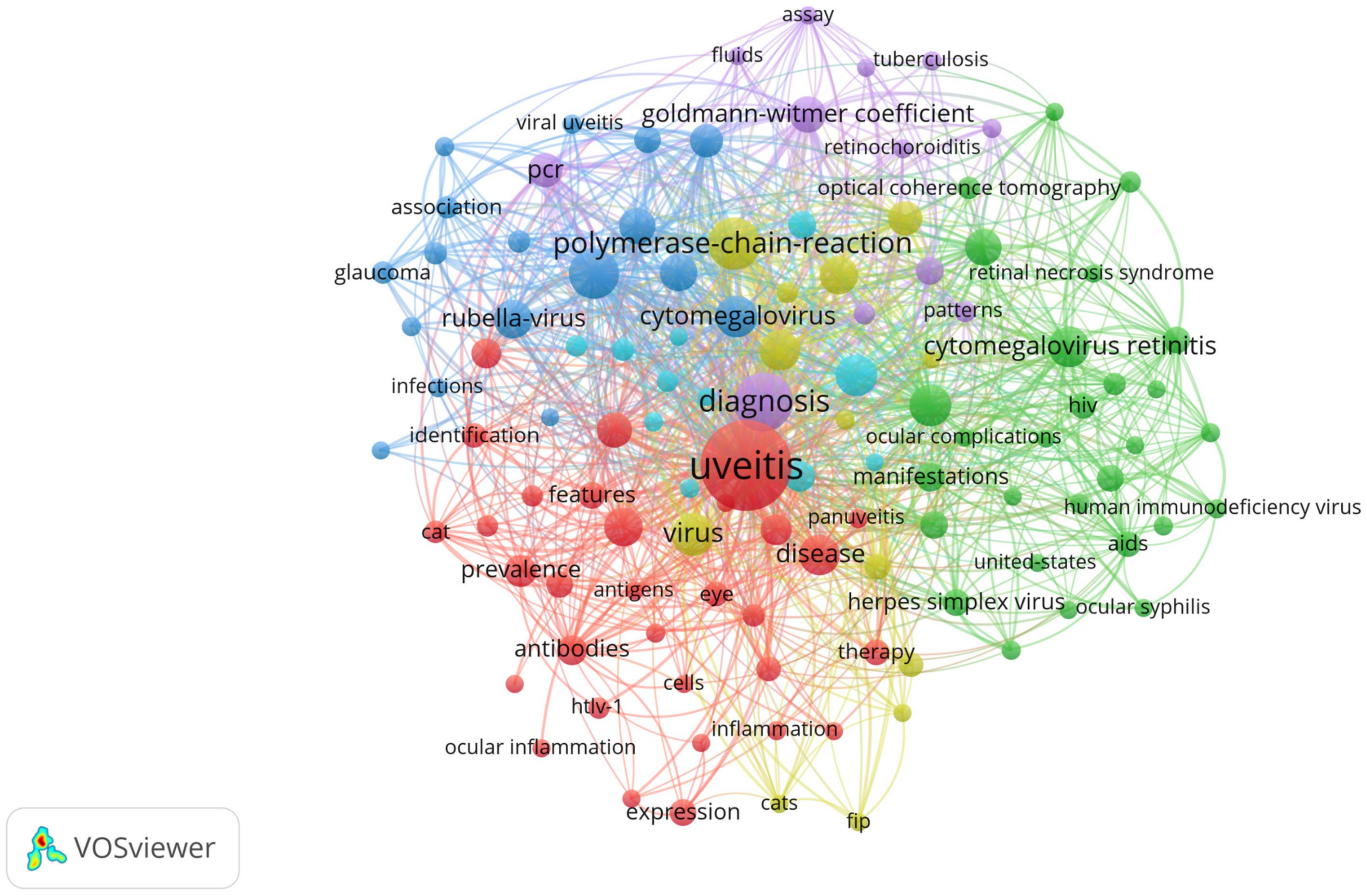
VOSviewer visualization of keywords from the top 100 highly cited articles in the field of virus and infectious uveitis.

VOSviewer software was utilized to generate a keyword co-occurrence network diagram, providing an intuitive and efficient means to identify the forefront and hotspots within a specific research field. VOSviewer extracted and clustered the top keywords in the present study, Figure 4 displays a visualization network map of top keywords in five clusters. In this figure, these node label indicate the keyword, and the size of each node refers to the frequency of the keywords. Lines connecting two nodes represent the co-occurrence relationship between those two keywords. All keywords were grouped into 5 clusters automatically by VOSviewer. The colors of 5 groups are purple (cluster 1), yellow (cluster 2), blue (cluster 3), red (cluster 4), and green (cluster 5). Cluster 1 in purple represented the diagnostic criteria of viral uveitis, Cluster 2 in yellow represented the diagnosis tool of viral uveitis, Cluster 3 in blue represented the clinical manifestations of viral uveitis, Cluster 4 in red represented the epidemiology of viral uveitis, Cluster 5 in green represented the etiology of viral uveitis. Recent hot spots and trends are reflected in these five aspects.

### Analysis of keywords

**Figure 5:**
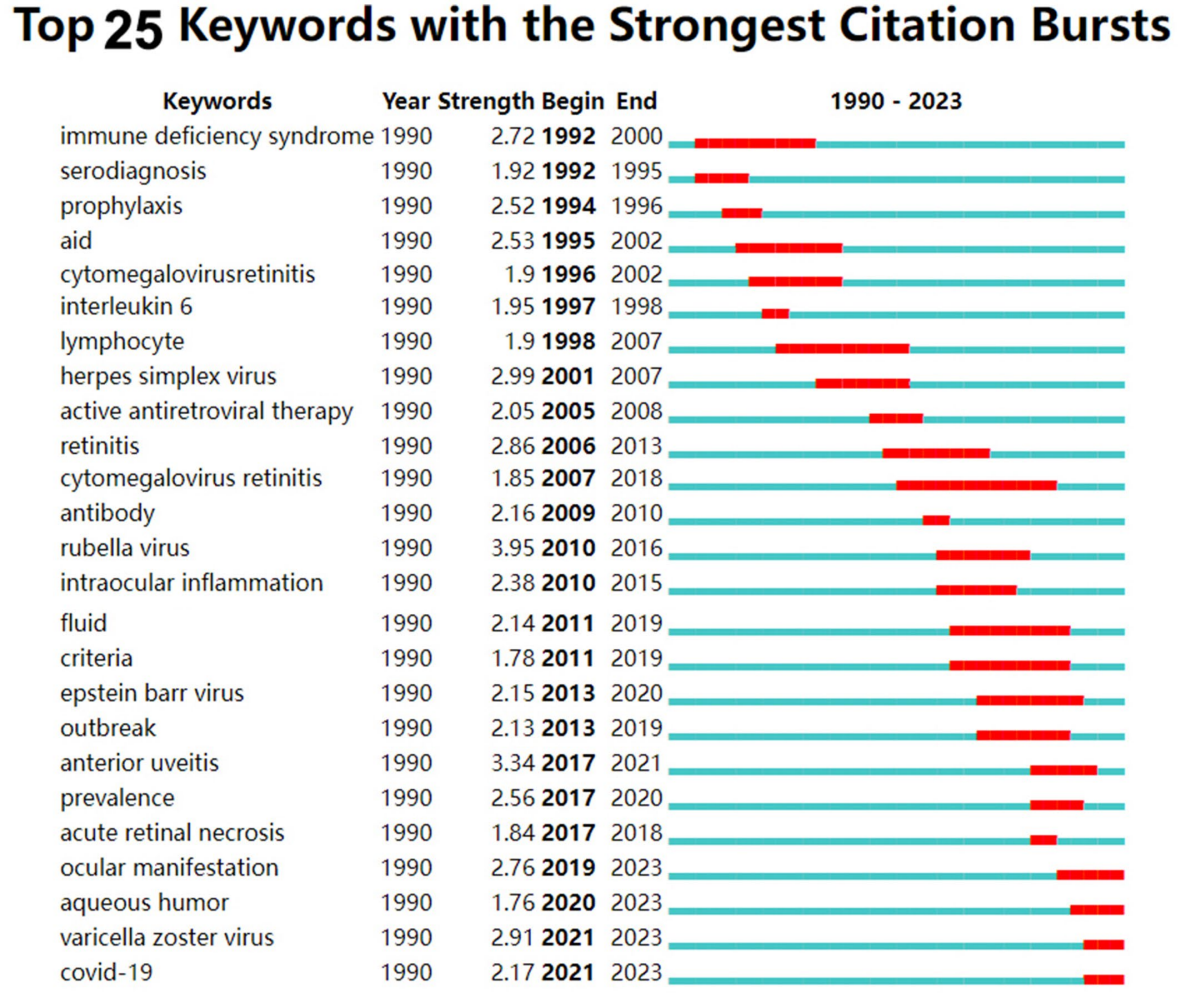
Top 25 keywords with the strongest citation bursts from the 281 cited articles in the field of virus and infectious uveitis.

Figure 5 shows the keyword highlighting diagram. The green line is the timeline, and the red segment on the green line is the burst detection, indicating the start year, end year and duration of the burst detection. The figure shows the annual changes in the top 25 most cited keywords for virus and infection uveitis in different periods. The results show that among these 30 prominent keywords, the earliest prominent keywords regarding virus types are immunodeficiency syndrome (duration 1992–2000), and cytomegalovirus retinitis appearing in 2007 (2007–2018), HSV appeared in 2001 (2001–2007), rubella virus appeared in 2010 (2010–2016), EBV appeared in 2013 (2013–2020), VZV appeared in 2021 (2021–2023), and new coronavirus appeared in 2021 (2021–2023). Among them, rubella virus has the highest intensity, which is 3.95; cytomegalovirus retinitis has the longest prominence span, lasting 11 years, indicating that it remains a subject of considerable concern. Regarding testing objects, the earliest prominent words were serology testing (1992–1995), and aqueous humor appeared in 2020 (2020–2023).

### Clinical and demographic characteristics of patients with suspected infectious uveitis

Table 1 reveals the characteristics and clinical data of all patients with suspected infectious uveitis. According to the inclusion and exclusion criteria, a total of 73 patients with suspected infectious uveitis, consisting 39 males and 34 females, corresponding to 73 eyes, were included. Males accounted for 53.42% (39/73), females accounted for 46.58% (34/73). The youngest patient was 6 years old and the oldest patient was 82 years old, and the average age was 49.05 ± 17.87 years old. Among these 73 samples, 33 were from the left eye, and 30 were from the right eye. The rest 10 were infected in both eyes, and only one sample was taken for testing.

**Table 1:** Characteristics and clinical data of all patients with suspected infectious uveitis.

Case number	Sex/age/affected eye	Intraocular fluid	Microorganism detected by mNGS	Culture of bacteria or fungi
1	M/26/L	Vit	HSV2, EBV	N
2	F/8/R	Vit	*Streptococcus oralis*	Y
3	M/6/R	Vit	*Streptococcus oralis*, *Streptococcus mitis*, *Abiotrophia defectiva*	Y
4	M/53/R	Aq	*Enterococcus faecalis*, Torque teno virus, mesovirus 1	Y, *Enterococcus faecalis*
5	F/60/R	Vit	Neoellipticus	N
6	M/57/L	Vit	*Streptococcus salivarius*	N
7	F/35/L	Vit	Puccinia fabae	Y
8	F/64/L	Vit	*Aspergillus oryzae*, Torque teno virus	Y
9	M/52/L	Vit	*Aspergillus fumigatus*	Y
10	M/28/R	Vit	*Ewingella americana*, *Serratia grimesii*, *Byssochlamys spectabilis*	Y
11	F/58/L	Vit	EBV	N
12	M/11/L	Aq	VZV	Y
13	M/68/B	Vit	*Candida albicans*, Torque teno virus	Y
14	F/73/L	Vit	*Rickettsia felis*	N
15	M/72/L	Vit	VZV, EBV	Y
16	M/65/R	Vit	*Staphylococcus aureus*	Y
17	F/56/R	Aq	EBV	N
18	M/27/L	Aq	VZV, EBV	N
19	M/51/R	Vit	*Candida albicans*, Human beta herpesvirus5	Y
20	M/76/R	Aq	VZV, *Mycobacterium abscessus*, *Candida parapsilosis*	N
21	F/54/L	Vit	*Acinetobacter baumannii*, HSV1	N
22	F/54/R	Aq	VZV, *Staphylococcus aureus*	N
23	F/35/R	Aq	*Penicillium*	Y
24	F/26/L	Aq	VZV, *Staphylococcus aureus*	N
25	M/55/R	Aq	*Acinetobacter pittii*, *Treponema pallidum*, *Staphylococcus cohnii*	N
26	M/64/L	Aq	*Staphylococcus lugdunensis*, *Staphylococcus aureus*	Y
27	M/41/R	Aq	*Pseudomonas aeruginosa*	Y
28	F/81/R	Aq	EBV	N
29	M/40/B	Aq	*Klebsiella pneumoniae*	Y
30	F/72/R	Vit	*Arthrobacter woluwensis*, Torque teno virus	Y
31	M/60/L	Aq	VZV	N
32	M/32/L	Vit	*Staphylococcus epidermidis*	Y
33	F/51/L	Aq	*Toxoplasma gondii*, *Streptococcus constellatus*, *Fusarium solani*	N
34	F/59/B	Vit	HSV2, EBV	N
35	M/68/B	Aq	Torque teno virus	Y
36	M/37/R	Aq	VZV	N
37	M/43/R	Aq	*Candida parapsilosis*	N
38	F/69/L	Aq	VZV	Y
39	M/43/B	Aq	Human papillomavirus 209	N
40	M/53/L	Aq	VZV	N
41	F/45/B	Aq	*Citrobacter fraudii*, Gamma papillomavirus 8	N
42	M/40/L	Aq	Mycorrhizal pine	N
43	F/58/L	Aq	Mycorrhizal pine, *Citrobacter brucei*, Beta papillomavirus 2	N
44	M/18/R	Aq	EBV	N
45	M/59/L	Aq	VZV	N
46	M/59/L	Vit	*Klebsiella pneumoniae*	Y
47	M/69/L	Vit	Torque teno virus	Y
48	F/59/R	Aq	VZV	N
49	F/38/R	Aq	Human beta herpesvirus7	N
50	F/26/L	Aq	*Klebsiella pneumoniae*, Torque teno virus	N
51	M/82/L	Aq	*Streptococcus pseudopneumoniae*, Torque teno virus	N
52	F/34/R	Aq	Human polyomavirus 5	N
53	F/59/L	Aq	VZV	N
54	F/69/L	Aq	*Staphylococcus aureus*, EBV, Torque teno virus	Y
55	F/43/L	Vit	None	Y
56	M/59/R	Vit	None	Y
57	M/26/L	Vit	None	N
58	F/57/R	Vit	None	N
59	M/59/R	Vit	None	N
60	M/43/L	Vit	None	Y
61	F/12/R	Aq	None	N
62	M/32/R	Vit	None	N
63	M/73/L	Vit	None	Y
64	M/47/L	Aq	None	N
65	F/53/R	Aq	None	N
66	F/50/B	Aq	None	N
67	F/36/L	Aq	None	N
68	M/59/B	Aq	None	N
69	M/29/R	Aq	None	N
70	F/36/R	Aq	None	N
71	F/67/B	Vit	None	Y
72	F/35/R	Vit	None	N
73	F/67/B	Vit	None	Y

mNGS, Metagenomic next-generation sequencing; M, male; F, female; R, right eye; L, left eye; B, both eyes; Aq, aqueous humor; Vit, vitreous humor; N, no; Y, yes; HSV, herpes simplex virus; EBV, Epstein–Barr virus; VZV, varicella zoster virus.

All the patients showed signs of panuveitis, including 14 cases complicated with retinal detachment. These patients were characterized by indications of infection, such as unresponsiveness to conventional steroid/immunosuppressive therapies or obvious retinal necrosis lesions identified during eye examinations. As shown in Table 1, intraocular fluids from 73 eyes suspected of infectious uveitis underwent mNGS testing, including 41 samples of aqueous humor and 32 samples of vitreous humor. Among these 29 samples sent for microbial culture, 23 samples detected pathogens by mNGS, while 6 samples did not detect pathogens by mNGS either. Only *Enterococcus faecalis* can be detected both by mNGS and microbial culture.

### Comparison of pathogens detection by mNGS and microbial culture

**Figure 6:**
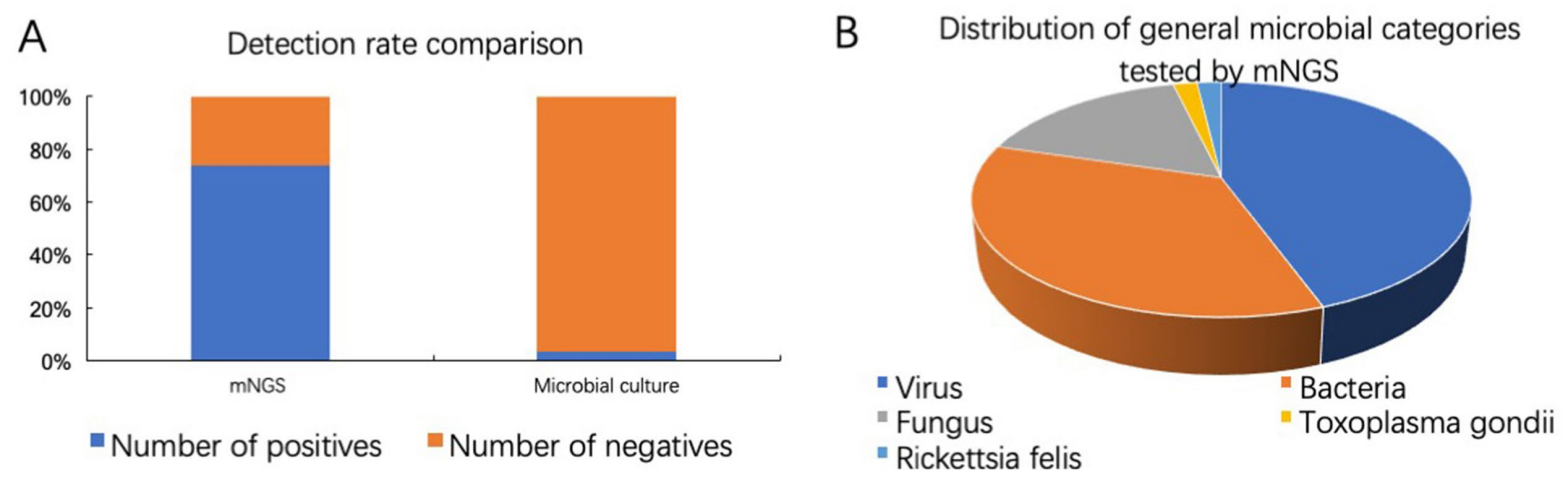
Comparison of pathogens detection by mNGS and microbial culture. **(A)** Detection rates of mNGS and microbiological culture. **(B)** Distribution of general microbial categories tested by mNGS.

As shown in Figure 6A, among 73 samples, 54 samples were tested positive by mNGS, yielding a positivity rate of 73.97%. Intraocular fluids from 29 samples were sent for microbiological culture at the same time, and only 1 sample was cultured positive (*Enterococcus faecalis*). mNGS offers unique advantages for detecting viruses that are challenging to culture. Among 54 positive samples tested by mNGS, 24 cases were mainly virus infected, 19 cases were mainly bacteria infected, 9 cases were mainly fungus infected, 1 case each for *Toxoplasma gondii* and *Rickettsia felis* (Figure 6B).

### mNGS pathogen detection results analysis

**Figure 7:**
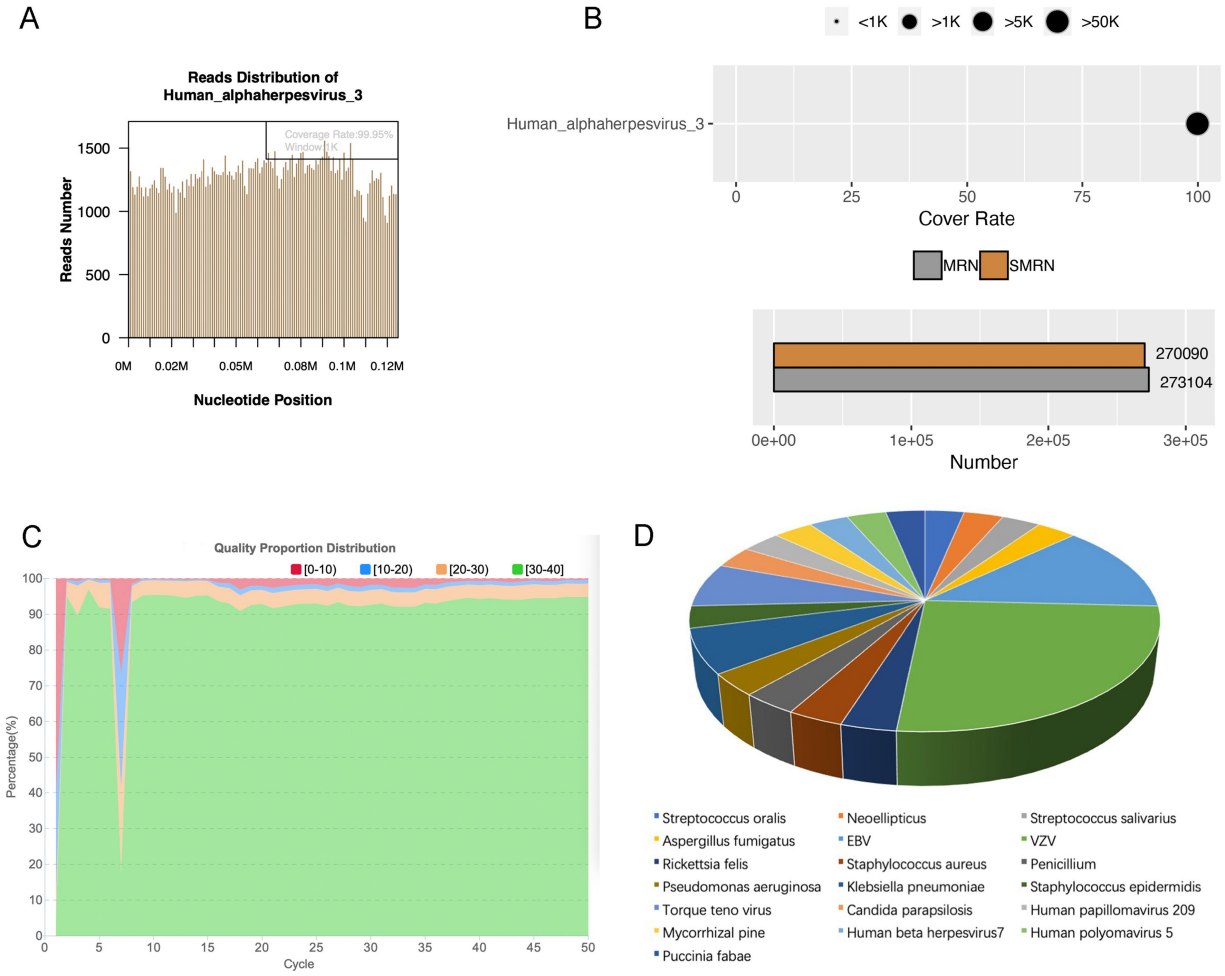
Single-pathogen infection detected by mNGS. **(A)** Pathogen genome coverage map. **(B)** Sequencing coverage and depth results. **(C)** Data quality control results. **(D)** Pathogen distribution in single-pathogen infection detected by mNGS.

We classified cases as single-pathogen infection when only one pathogen was detected. 31 cases were single-pathogen infection detected by mNGS, of which virus was the main pathogen, detected in 17 eyes. Bacteria were detected in 7 eyes, fungi were detected in 6 eyes, and *Rickettsia felis* was detected in one patient. For instance, in a patient, unbiased mNGS of intraocular fluids detected 270,090 sequence reads associated with VZV, with a relative abundance of 99.99%. This achieved a coverage of 99.95% of the VZV virus genome (Figures 7A,B). Figure 7C demonstrated the quality control measures applied to the mNGS data, indicating high reliability. As shown in Figure 7D, among 31 single-pathogen infection, the most frequently identified pathogen was as follows: 8 cases of VZV (8/31, 25.81%), 4 cases of EBV (4/31, 12.90%), 2 cases of *Klebsiella pneumoniae* (2/31, 6.45%), and 2 cases of Torque teno virus (2/31, 6.45%).

Interestingly, we found that 23 patients had 2 or more microorganisms in the intraocular fluid, including viral mixed existing, bacterial mixed existing, bacterial mixed existing, viral and fungal mixed existing, among which viruses were detected the most. Table 2 shows the status of coexisting microorganisms and their genus-specific read number tested by mNGS. It is evident that coexisting-infections may occur in patients with infectious uveitis, indicating the necessity for combination drug therapy during treatment. As shown in Table 2, out of the 54 positive cases, 23 cases exhibited coexisting-infections. Among the 32 coexisting microorganisms, viruses were detected the most, 18 times, accounting for 56.25%. There were 4 cases of viral mixed infection, 4 cases of bacterial mixed infection, 6 cases of bacterial and viral mixed infection, 4 cases of fungal and viral mixed infection, 1 case of bacterial and fungal mixed infection, and 2 mixed viral, bacterial, and fungal infections. The most common coexisting microorganisms were Torque teno virus, accounting for 7 cases (7/32, 21.88%), followed by EBV, with 4 cases (4/32, 12.50%). Although mNGS identifies potential pathogens, physician assessment is necessary to determine clinical significance.

**Table 2:** Coexisting microorganisms tested by mNGS.

Case number	Microorganism detected by mNGS	Genus-specific read number (reads)
1	HSV2, EBV	11, 4
3	*Streptococcus oralis*, *Streptococcus mitis*, *Abiotrophia defectiva*	158,134, 3,738, 775
4	*Enterococcus faecalis*, Torque teno virus, mesovirus 1	16,746, 9, 5
8	*Aspergillus oryzae*, Torque teno virus	72, 4
10	*Ewingella americana*, *Serratia grimesii*, *Byssochlamys spectabilis*	653,216, 146,208, 122
13	*Candida albicans*, Torque teno virus	32, 14
15	VZV, EBV	24, 7
18	VZV, EBV	79,351, 7
19	*Candida albicans*, Human beta herpesvirus 5	17,270, 1,496,114
20	VZV, *Mycobacterium abscessus*, *Candida parapsilosis*	53,630, 7, 4
21	*Acinetobacter baumannii*, HSV1	7,770, 5
22	VZV, *Staphylococcus aureus*	199,700, 22
24	VZV, *Staphylococcus aureus*	24,058, 39
25	*Acinetobacter pittii*, *Treponema pallidum*, *Staphylococcus cohnii*	11,401, 517, 1,033
26	*Staphylococcus lugdunensis*, *Staphylococcus aureus*	395, 66
30	*Arthrobacter woluwensis*, Torque teno virus	30,014, 8
33	*Toxoplasma gondii*, *Streptococcus constellatus*, *Fusarium solani*	776, 6, 4
34	HSV2, EBV	4, 3
41	*Citrobacter fraudii*, Gamma papillomavirus 8	161, 3
43	Mycorrhizal pine, *Citrobacter brucei*, Beta papillomavirus 2	1,051, 879, 5
50	*Klebsiella pneumoniae*, Torque teno virus	57, 7
51	*Streptococcus pseudopneumoniae*, Torque teno virus	42,830, 19
54	*Staphylococcus aureus*, EBV, Torque teno virus	70, 30, 5

mNGS, Metagenomic next-generation sequencing; HSV, herpes simplex virus; EBV, Epstein–Barr virus; VZV, varicella zoster virus.

## Discussion

The present study conducted a bibliometric analysis of 281 cited studies on infectious uveitis and viruses retrieved from WoSCC database. Our findings suggest that studies of infectious uveitis and viruses have undergone a significant growth in the past 3 years, 2021 having the highest annual growth. We found that the majority of published articles are concentrated around a few countries. The most productive authors were noted to be affiliated with institutions in the United States and Japan, while the United States has the most cooperative relationships with other nations. Furthermore, we have identified the most used terminologies from the articles from the author’s keywords and categorized them into five different groups. We found that the focus of research on infectious uveitis viruses has a shift in the predominant viral types, and a shift from serodiagnosis to intraocular fluid testing. In addition, the co-occurrence analysis revealed that the diagnostic criteria of viral uveitis, and the diagnosis tool of viral uveitis, the clinical manifestations of viral uveitis, epidemiology of viral uveitis, the etiology of viral uveitis may be predictive indicators of where the field is evolving toward and may be referenced by prospective scholars. Therefore, to understand the currently dominant virus species, we investigated the application and detection results of mNGS in suspected cases of infectious uveitis in China.

Through the co-occurrence analysis, it can be found that the current attention on virus and infectious uveitis mainly focuses on (1) the diagnostic criteria of viral uveitis. Standardization of Uveitis Nomenclature (SUN) Working Group proposed a great deal of classification criteria of viral uveitis, such as cytomegalovirus anterior uveitis (Standardization of Uveitis Nomenclature Working Group, 2021a), Herpes Simplex Virus Anterior Uveitis (Standardization of Uveitis Nomenclature Working Group, 2021b), Varicella Zoster Virus Anterior Uveitis (Standardization of Uveitis Nomenclature Working Group, 2021c). (2) THE diagnosis tool of viral uveitis. The PCR analysis of intraocular fluid in patients suspected of infectious uveitis holds significant importance for both confirming diagnosis and guiding treatment adjustments (Fekri et al., 2023). mNGS emerges as a highly sensitive, unbiased, and comprehensive method, showing considerable promise in identifying the causative pathogens in intraocular infections (Doan et al., 2017). (3) The clinical manifestations of viral uveitis. Viral anterior uveitis may present as either granulomatous or non-granulomatous uveitis, often accompanied by keratic precipitates of varying characteristics. This can occur with or without endotheliitis, leading to increased intraocular pressure (Feng et al., 2023). (4) epidemiology of viral uveitis. Concerning the localization of inflammation in viral uveitis, anterior involvement predominates and has been documented in a range of 54–91% across diverse global studies (Radosavljevic et al., 2022). Bro T reported a retrospective, cohort study in Sweden, finding that probable herpetic uveitis cases based on clinical diagnosis of herpes simplex virus (HSV) or VZV account for 4.9% of all uveitis cases (Bro and Tallstedt, 2020). Another Retrospective, population-based cohort study reported that herpetic uveitis account for 8% of all uveitis cases (Acharya et al., 2013). (5) Etiology of viral uveitis. Viral etiologies of uveitis include HSV, VZV, cytomegalovirus (CMV), EBV, rubella virus, human T-cell leukemia virus type 1 (HTLV-1) virus (Tugal-Tutkun et al., 2010; Wang et al., 2012; Lachmann et al., 2018).

Keyword highlighting diagram reveal the evolution of each keyword over time. Through the analysis of keywords, we found that in the field of virus and infectious uveitis advances, the focus of research has shifted in two directions. We have noted a change in the dominant viral strains over time. While previous studies mainly concentrated on cytomegalovirus and HSV, recent years have witnessed a growing emphasis on EBV, VZV, and COVID-19. Hence, medication adjustments are necessary based on the specific virus type. Among these 25 prominent keywords, rubella virus exhibits the highest intensity at 3.95, spanning from 2010 to 2016. According to a report by S R Rathinam et al., among 15,221 patients with uveitis, herpetic anterior uveitis emerged as a common cause of infectious uveitis, constituting 5–10% of all uveitis cases (Rathinam and Namperumalsamy, 2007). In 2002, Elisabetta Miserocchi et al. published a retrospective study in *ophthalmology*, a leading journal in ophthalmology, which compared the clinical characteristics and prognosis of patients with uveitis caused by HSV and VZV. The study highlighted that uveitis induced by herpes simplex virus tends to have a relatively mild course but is prone to relapse, with a low likelihood of posterior pole inflammation (Miserocchi et al., 2002). This research represents the first direct comparison between patients with HSV and VZV uveitis, thus it has a high number of citations and has important clinical significance for the clinical differential diagnosis of HSV. The keyword that has been highlighted for the longest time is cytomegalovirus retinitis. Cytomegalovirus retinitis often occurs in patients with low immune function, such as newborns, bone marrow or organ transplant recipients, and AIDS patients (Saidel et al., 2005; von Hofsten et al., 2016). Meanwhile, treatment of cytomegalovirus retinitis remains a clinical challenge. Patients require anti-cytomegalovirus maintenance therapy for several months, and resistance often develops. Therefore, research on cytomegalovirus retinitis continues to receive high attention (Port et al., 2017). COVID-19 has emerged as a research focal point over the past 3 years, suggesting a heightened attention or prevalence of viral uveitis due to the onset of the coronavirus pandemic. As recently reported, uveitis may manifest following administration of the new coronavirus vaccine (Zhong et al., 2022).

The second research hotspot is the shift from serodiagnosis to intraocular fluid testing. Since the manifestations of viral infection in the eye often vary depending on pathogenicity and autoimmunity, the standardization of eye signs is also extremely important. Aqueous humor testing is becoming increasingly crucial in diagnosing uveitis due to its minimal invasiveness. Tests involving the detection of cytokines like interleukin 6, interleukin 10 (Errera et al., 2022), and PCR detection of pathogenic nucleic acids hold significant clinical relevance in uveitis diagnosis (Gozzi et al., 2022). In October 2023, *Cell* published a report on integrating proteomic analysis of aqueous humor with artificial intelligence to assess the staging and prognosis of eye diseases, including uveitis. This suggests that aqueous humor testing will become the focus and frontier of uveitis research in the future (Wolf et al., 2023).

In our clinical study, the positive rates of mNGS and culture were 73.97% (54/73) and 3.45% (1/29), respectively, which were all confirmed by other laboratory examinations or effective diagnostic treatment. mNGS demonstrates significant efficacy in detecting bacterial, viral, fungus, *Toxoplasma gondii*, *Rickettsia felis*, and mixed infections. The higher detection rate is mainly due to the non-culture dependence and unbiased nature of mNGS diagnosis of pathogens, making the detection results less affected by specific preparation and handling of different types of pathogens (Simner et al., 2018). Additionally, mNGS offers a faster detection speed compared to traditional culture methods (Pendleton et al., 2017). Among 54 positive samples tested by mNGS, 24 cases were mainly virus infected, 19 cases were mainly bacteria infected, 9 cases were mainly fungus infected, 1 case each for *Toxoplasma gondii* and *Rickettsia felis*. These findings suggest that viruses are one of the main pathogens of infectious uveitis.

In our study, the most common pathogens for single-pathogen infection were VZV (8/31, 25.81%), EBV (4/31, 12.90%), *Klebsiella pneumoniae* (2/31, 6.45%), and Torque Teno Virus (2/31, 6.45%). In the past, virus types were predominantly focused on cytomegalovirus and HSV. In a clinical study conducted in southern Taiwan Province, the prevalence of virus-associated anterior uveitis among cases not associated with HLA was investigated. PCR primers targeting HSV, VZV, CMV, and EBV viruses were employed for detection across a cohort of 102 eyes. The findings revealed positivity for herpes virus in 42 eyes, among which 9 were attributed to HSV-related anterior uveitis, 5 to VZV-related anterior uveitis, 27 to CMV-related anterior uveitis, and 1 to EBV-associated anterior uveitis (Hsiao et al., 2019). In a study of the Italian population, HSV was the most common cause of viral anterior uveitis, accounting for 83.3%, followed by VZV and CMV (Miserocchi et al., 2014). Engelhard et al. found in USA, the most common pathogen in viral anterior uveitis was HSV, followed by VZV (43.6%) and CMV (2.6%) (Engelhard et al., 2015). However, there has been a shift in the main virus types, indicating potential changes in our living environment. We also observed multiple instances of coexisting microorganisms. The most prevalent coexisting microorganisms were Torque Teno Virus (21.88%) and EBV (12.50%). Torque Teno Virus has been detected in healthy people, however, it can be activated when the patient is immunosuppressed (Sajiki et al., 2023). Recent study has also shown that EBV often causes co-infection (Silpa-Archa et al., 2022). Hence, when encountering an ineffective antibacterial drug, it’s essential to consider the possibility of multiple infections and opt for combination therapy. Multiple infections, errors, or secondary infections—ultimately, it depends on the clinician’s judgment.

However, previous detection methods have certain shortcomings. Conventional microbial culture remains the gold standard for diagnosing intraocular bacterial and fungal infections. However, the identification of pathogens requires a few days to weeks (Zhu et al., 2022). PCR can be used to detect viral infections and has high sensitivity and specificity when we have the suspicion of a specific pathogen, but its limitation is that it can only detect specific target sequences and cannot provide information on the overall microbial community. Based on the above, due to limited detection methods, previous research has mainly focused on bacterial and fungal endophthalmitis, but there has been little research on viral uveitis. mNGS, as an efficient, unbiased and rapid detection method, shows unique advantages in pathogenic diagnosis. mNGS analysis can provide information on the overall microbial community, with high resolution and coverage. However, its disadvantages include complex operation, high demand for data analysis, inability to determine microbial activity and high costs. In the future, we anticipate advancements in detection methods and options, new discoveries in the field of infectious uveitis, and improved services for its diagnosis and treatment.

### Limitations of this research

This study is a single center study with specific regional characteristics, and the population is concentrated in Zhejiang Province, China. We will conduct a multi-center research in the future.

## Conclusion

Viruses exhibit a significant correlation with infectious uveitis, as evidenced by thorough bibliometric and clinical data analysis. mNGS has a high rate in identifying different types of pathogens in infectious uveitis by minute volumes of intraocular fluid samples. This contributes to the accumulation of clinical data on infectious uveitis and empirical medication for infectious uveitis.

## Data Availability

The original contributions presented in the study are publicly available. This data can be found here: NCBI BioProject (https://www.ncbi.nlm.nih.gov/bioproject/), accession number: PRJNA1301882.

## Footnotes

^1^ ftp://ftp.ncbi.nlm.nih.gov/genomes/
